# Potential Circumferential Bone Engagement following Tooth Extraction in the Posterior Mandible: Computed Tomography Assessment

**DOI:** 10.3390/medicina57090874

**Published:** 2021-08-26

**Authors:** Yafit Hamzani, Emran Yassien, Liad Moskovich, Talia Becker, Gavriel Chaushu, Bahaa Haj Yahya

**Affiliations:** 1 Department of Oral and Maxillofacial Surgery, Rabin Medical Center—Beilinson Hospital, Petach Tikva 4941492, Israel; becktalia@gmail.com (T.B.); gabi.chaushu@gmail.com (G.C.); 2 Department of Oral and Maxillofacial Surgery, The Maurice and Gabriela Goldschleger School of Dental Medicine, Tel Aviv University, Tel Aviv 6139001, Israel; Aboalyassien@gmail.com; 3 Department of Oral and Maxillofacial Surgery, Galilee Medical Center, Nahariya 2210001, Israel; dr.moskovichliad@gmail.com; 4 Oral and Maxillofacial Clinic, Herzliya 4672211, Israel; bahaa.hag@gmail.com

**Keywords:** immediate implant placement, posterior mandible, cone beam computed tomography, circumferential engagement, implant length

## Abstract

*Background and Objectives*: Immediate implant placement (IIP) is a popular surgical procedure with a 94.9–98.4% survival rate and 97.8–100% success rate. In the posterior mandible, it poses a risk of injury to adjacent anatomical structures if the implant engages apical bone. This study sought to assess the implant dimensions that allow for circumferential bone engagement at each position in the posterior mandible without additional apical drilling. *Materials and Methods*: An observational, cross-sectional study design was used. The pre-extraction cone beam computed tomography scans of 100 candidates for IIP were analyzed. Measurements of each root of the posterior mandibular second premolar, first molar, and second molar were taken from three aspects: buccolingual, mesiodistal, and vertical. Two-sided *p* values < 0.05 were considered statistically significant. *Results*: A total of 478 mandibular teeth and 781 roots were assessed. Based on Straumann^®^ BLX/BLT implant-drilling protocols, predicted rates of radiological circumferential engagement (RCE) were 96% for implants 5 mm in diameter in the second premolar root position; 94% for implants 4.0–4.2 mm in diameter in the first molar root position; and 99% for implants 4.5–4.8 mm in diameter in the second molar root position. Corresponding rates of achieving an available implant length (AIL) of 10 mm were 99%, 90%, and 86%. Patients <40 years old were at higher risk of lower RCE and lower AIL (*p* < 0.005) than older patients for all roots measured. *Conclusions*: The high primary stability prediction rates based on the calculation of RCE and AIL support the use of IIPs without further apical drilling in the posterior mandible in most cases.

## 1. Introduction 

The insertion of dental implants on the same day of tooth extraction, termed immediate implant placement (IIP), is a popular surgical procedure [1]. Survival rates range from 94.9% to 98.4% [2,3,4,5,6], and success rates range from 97.8% to 100% [7,8]. IIP is in high demand by both surgeons and patients for the non-esthetic zone because it reduces the number of surgical interventions and allows for earlier initiation of prosthodontic therapy [8]. 

There are several acceptable approaches to achieve bone engagement and primary stability in posterior mandibular IIP [2,9,10,11,12]. One of them is vertical native bone anchorage. However, this requires the dental surgeon to perform additional apical drilling, as described in recent studies [13,14,15]. Using this approach, researchers found that the risk of injuring the inferior alveolar nerve (IAN) during implant insertion was 48% in second premolars, 32% in first molars, and 64% in second molars [13]. Froum et al. [14] reported even higher rates of 73% in second molars, 65% in second premolars, and 53% for first molars. This was true even when implants were stabilized with the apical and/or lateral bone [14] according to the general consensus requirement of 6 mm of native bone apical to the socket: 4 mm for apical anchorage [12,16,17,18,19] and a 2 mm safety zone [13,20]. 

Lin et al. [15] showed that the risk of IAN injury was 3.8-fold higher in the mandibular second molar than the mandibular second premolar. The reported risk of lingual plate perforation was also very high: 70% in first molars and 76% in second molars [13]. One study reported lingual plate perforation with severe hemorrhage in the floor of the mouth in 21/25 patients (84%) undergoing IIP, leading to emergency intubation in 17 [21]. All these studies concluded that in the posterior mandible, IIP based on native apical bone anchorage is dangerous and may impair adjacent anatomical structures with additional complications [13,14,15].

To overcome this obstacle, several groups described alternative methods based on stabilization of the implant with the circumferential socket walls [12,22]. In these approaches, the morphology of the molar extraction socket has a crucial impact on IIP circumferential engagement and stability, and elements influencing the morphology need to be taken into account, including tooth width at the cement–enamel junction in the buccolingual and mesiodistal aspects, root length, trunk length, and degree of divergence of the roots [12]. Smith and Tarnow [12] classified the molar extraction sockets from types A to C by amount of septal bone available for stabilization of the IIP. Another potential way to achieve primary stability is to engage the available bone with implants that are wider and shorter than the extracted root [23,24]. The aim of the present study was to verify, based on cone beam computed tomography (CBCT) scans, the implant dimensions that allow for circumferential bone engagement without additional apical drilling in each position in the posterior mandible. Factors that may contribute to circumferential bone engagement were further assessed. 

## 2. Materials and Methods

### 2.1. Setting and Design

The study was conducted in the department of oral and maxillofacial surgery of a tertiary medical center from July 2018 to January 2019. The study sample consisted of CBCT scans of adult patients (age > 18 years) with no history of chemotherapy or radiation to the jaws who were referred for posterior mandible dental implant placement. Only scans including at least two of the following posterior mandibular teeth were eligible for the study: mandibular second premolar, first molar, and second molar [13,14]. Of the 250 scans that met these criteria, 2 groups of 100 scans each were generated by “block” randomization method. One group consisting of 100 scans was chosen for review in the study, as in two similar studies [13,14]. Sample size and statistical power were not calculated since the current study was based on CBCT scans and in a corelated way to two similar studies [13,14]. The study was approved by the Helsinki Committee of Rabin Medical Center (approval number 0396-16-RMC; 19 August 2020).

### 2.2. Procedure

On each CBCT scan, an oral and maxillofacial resident and a dentist (Y.H, E.Y) independently measured each root of the mandibular second premolar, first molar, and second molar from 3 aspects: buccolingual, mesiodistal, and vertical. Thereafter, an oral and maxillofacial surgeon (B.H.Y.) re-examined 18 of the scans selected at random [14]. 

### 2.3. Measurements

**Figure 1:**
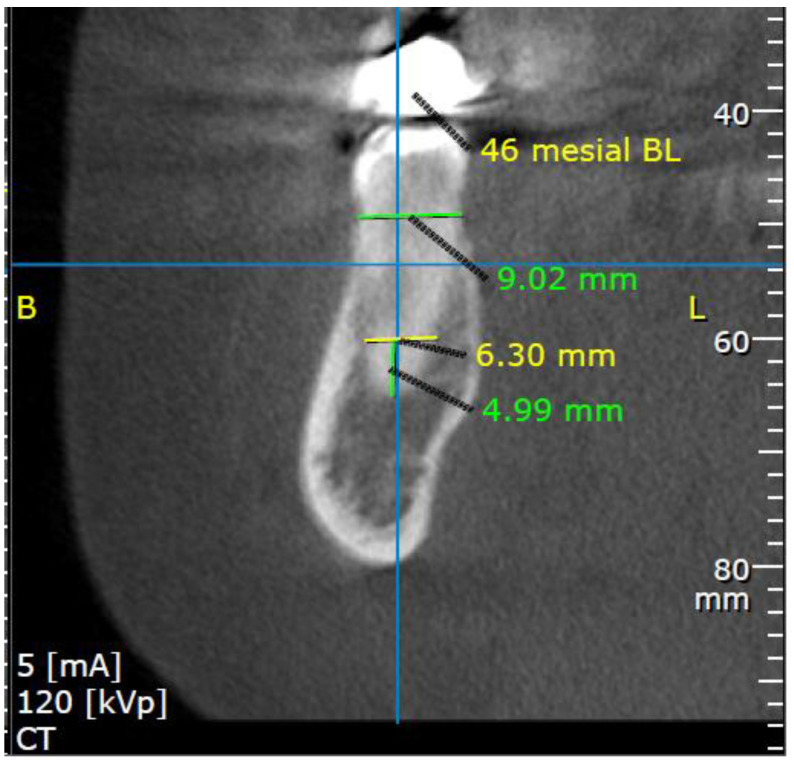
Buccolingual (BL) aspect measurements of right first molar, assessed in 2 different points, referred to tooth long axis: (1) A—5 mm coronal to the apices (4.99 mm from the apex; green line, the BL width is 6.3 mm; yellow line), (2) X- alveolar crest level (BL width is 9.02 mm; green line). CBCT para-axial cross section. B-buccal; L-lingual.

All measurements were evaluated separately using On Demand 3D software, version 1 (Cybermed Inc., Tustin, CA, USA). Buccolingual measurements were made on a para-axial cross-section slice representing the center of the tooth from 2 different points referred to the tooth’s long axis: 5 mm coronal to the apices (A point) and at the alveolar crest level (X point) (Figure 1).

**Figure 2:**
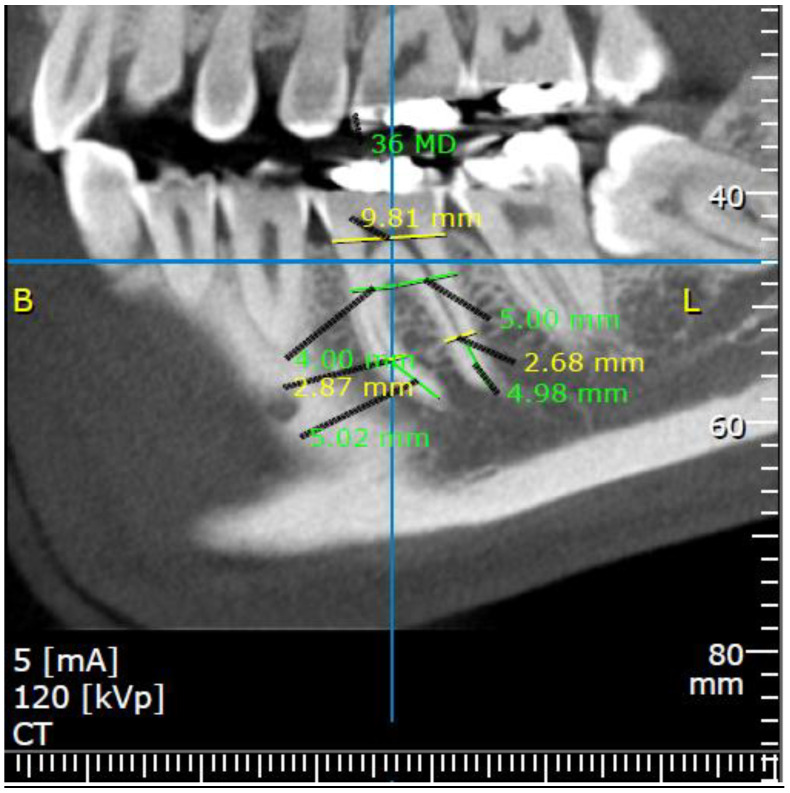
Mesiodistal (MD) aspect measurements of left first molar, assessed in 3 different points, referred to tooth long axis: (1) A—5 mm coronal to the apices (mesial root: 5.02 mm from the apex; green line, the MD width is 2.87 mm; yellow line); (distal root: 4.98 mm from the apex; green line, the MD width is 2.68 mm; yellow line), (2) F—at furcation level (MD width is 4 mm for mesial root and 5 mm for distal root), (3) X—alveolar crest level (MD width is 9.81mm). CBCT para-axial sagittal section.

Mesiodistal measurements were made on a para-sagittal cross-section slice representing the center of the tooth from 3 different points referred to the long axis: 5 mm coronal to the apices (A point), at the furcation level (F point), and at the alveolar crest level (X point) (Figure 2). 

**Figure 3:**
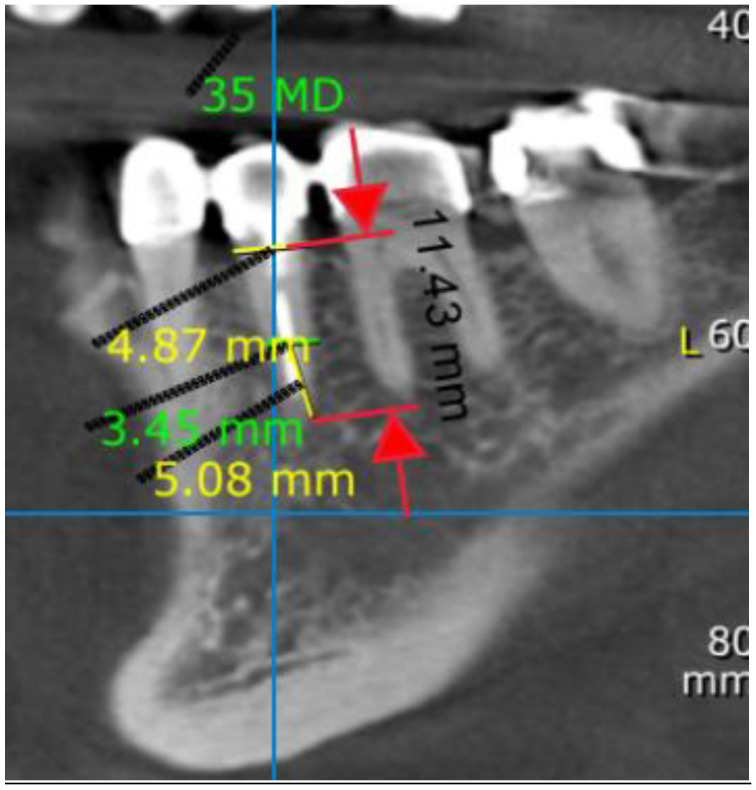
Vertical aspect of left second premolar, measured from apex to alveolar crest level; 11.43 mm in black. Mesiodistal (MD) aspect measurements, assessed in 2 points, referred to the long axis: (1) A—5 mm coronal to the apices (5.08 mm from the apex; yellow line, MD width is 3.45 mm; green line), (2) X—alveolar crest level (MD width is 4.87 mm; yellow line). CBCT para-axial sagittal section.

Vertical measurements were made on a para-axial cross-section slice representing the center of the tooth by tracing a vertical line from the most inferior point of the apices to the alveolar crest level (Figure 3). 

**Figure 4:**
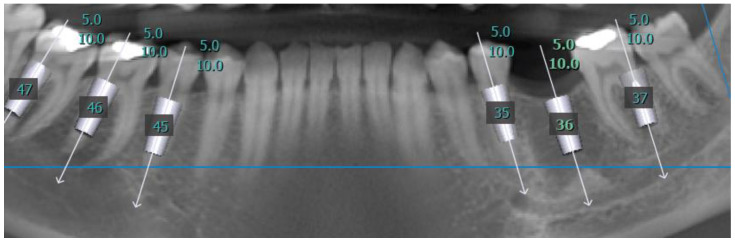
Virtual implant placement; positioning of 3 dental implants of 5 mm diameter and 10 mm long at each side of the mandibular sites: second premolar, distal root of first molar, distal root of second molar. CBCT panoramic reconstruction.

**Figure 5:**
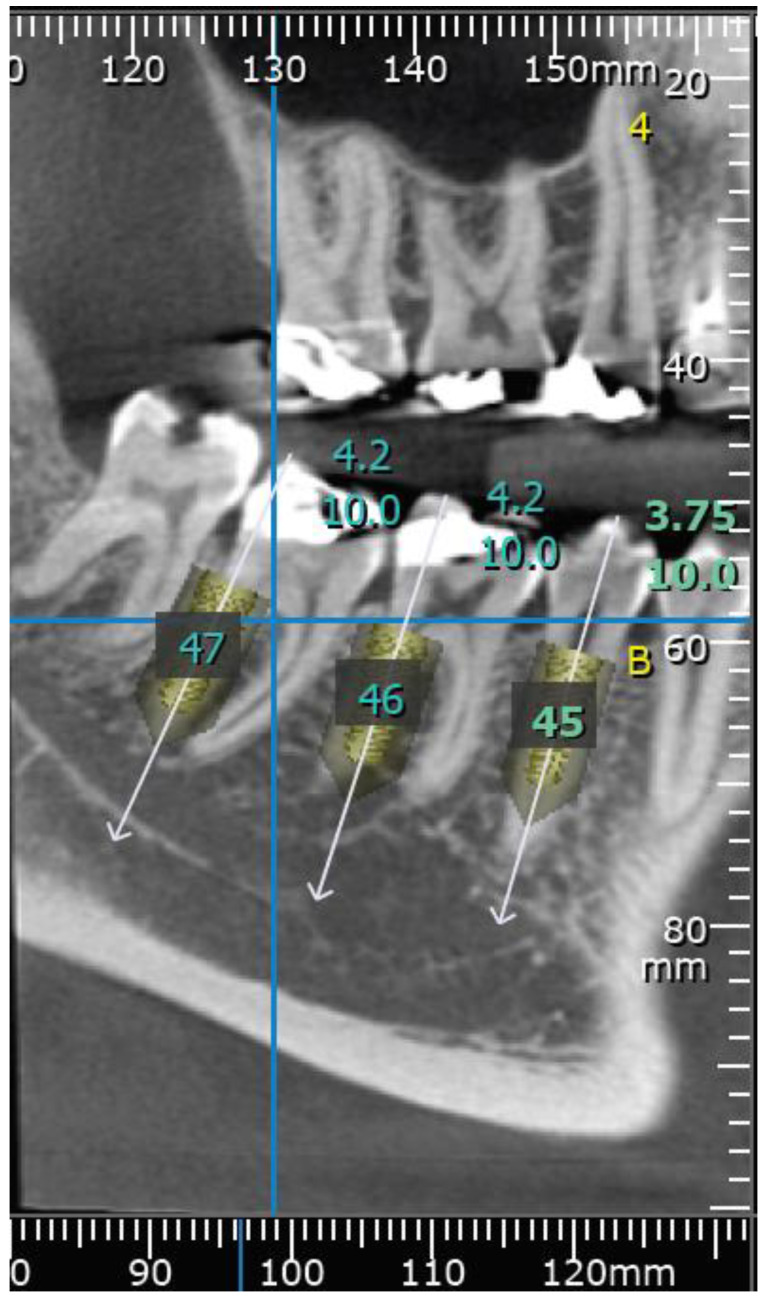
Virtual implant placement of 3 dental implants at right posterior mandibular sites: second premolar (3.75 mm diameter and 10mm length), distal root of first molar, and distal root of second molar (4.2 mm diameter and 10mm length each). CBCT para-axial sagittal section.

The data were recorded for each tooth and each root. Thereafter, virtual implant placement was used to verify the results and validate the measurements (Figure 4 and Figure 5).

In addition, dental implants of different diameters and lengths were virtually positioned in each root using On Demand 3D software. The predicted prevalence rate of radiological circumferential engagement (RCE) in the mesiodistal aspect was calculated on the basis of the Straumann^®^ bone level (BLX) and bone level tapered (BLT) implant-drilling protocols. The final drill diameter for each implant was measured, and RCE values were determined accordingly, as detailed in Table 1. Rates at which there was sufficient available implant length (AIL) without passing the apex were evaluated for implants 6 mm, 8 mm, and 10 mm long, as detailed in Table 2, according to the same Straumann^®^ drilling protocols

The mean mesiodistal distance at F point for each root type ranged from 3.86 mm for the mesial roots of the right second molar to 4.76 mm for the distal roots of the right first molar. The mean (SD) distance values at F point for each tooth type were 4.41 mm (0.56) for first molars and 4.32 mm (0.6) for second molars (Table 1). Points: A—5mm coronal to the apices; F- furcation level; X-alveolar crest level.

The mean AIL prevalence rates for a 10 mm-long implant were 99%, 90%, and 86% in the second premolar, first molar, and second molar root positions, respectively. Corresponding values for an 8 mm long implant were 100%, 98%, and 97%. For implants 6mm long, the mean rate was 100% for all tooth types (Table 2). X point—alveolar crest level. 

### 2.4. Patient Characteristics

The effect of the measured factors on outcome was assessed according to patient demographic data derived from the medical files. 

### 2.5. Statistical Analysis

Statistical analysis was generated using SAS software, version 9.4. Continuous variables are presented by mean and standard deviation (SD), and categorical variables, by number and percent. Student t-test and Wilcoxon signed-rank test were used to compare continuous variables between groups; for categorical variables, we used Fisher’s exact test (for 2 values) or chi-squared test (for more than 2 values). Two-sided *p* values < 0.05 were considered statistically significant. 

## 3. Results 

### 3.1. Patients

The study included 100 CBCT scans of 100 patients, 51 female and 49 males, of mean (SD) age 39.7 (15.1) years. The youngest patient was 18 years old, and the oldest was 88 years old; 63 patients were less than 40 years old and 37 were older. A total of 478 mandibular teeth (781 roots) were assessed: 175 (36.6%) second premolars (175 roots), 147 (30.7%) first molars (294 roots), and 156 (32.7%) second molars (312 roots). Inter-rater reliability (kappa coefficient) was 0.84. The distribution of the teeth/roots by side, patient age, and patient sex is shown in Table 3.

### 3.2. Distance Measurements

#### 3.2.1. Mesiodistal Aspect

The mean mesiodistal distance at a point for each root type ranged from 2.74 mm for the mesial roots of the left first molar to 3.66 mm for the mesial roots of the second premolar. The mean (SD) distance values at a point for each tooth type were 3.64 mm (0.51) for second premolars, 2.98 mm (0.5) for first molars, and 3.11 mm (0.5) for second molars (Table 1). 

The mean mesiodistal distance at X point for each tooth type ranged from 5.37 mm for the right second premolar to 9.25 mm for both roots of the left second molar. The mean (SD) distance values at X point for each tooth type were 5.41 mm (0.68) for second premolars, 9.14 mm (0.71) for first molars, and 9.19 mm (0.58) for second molars (Table 1). 

**Figure 6:**
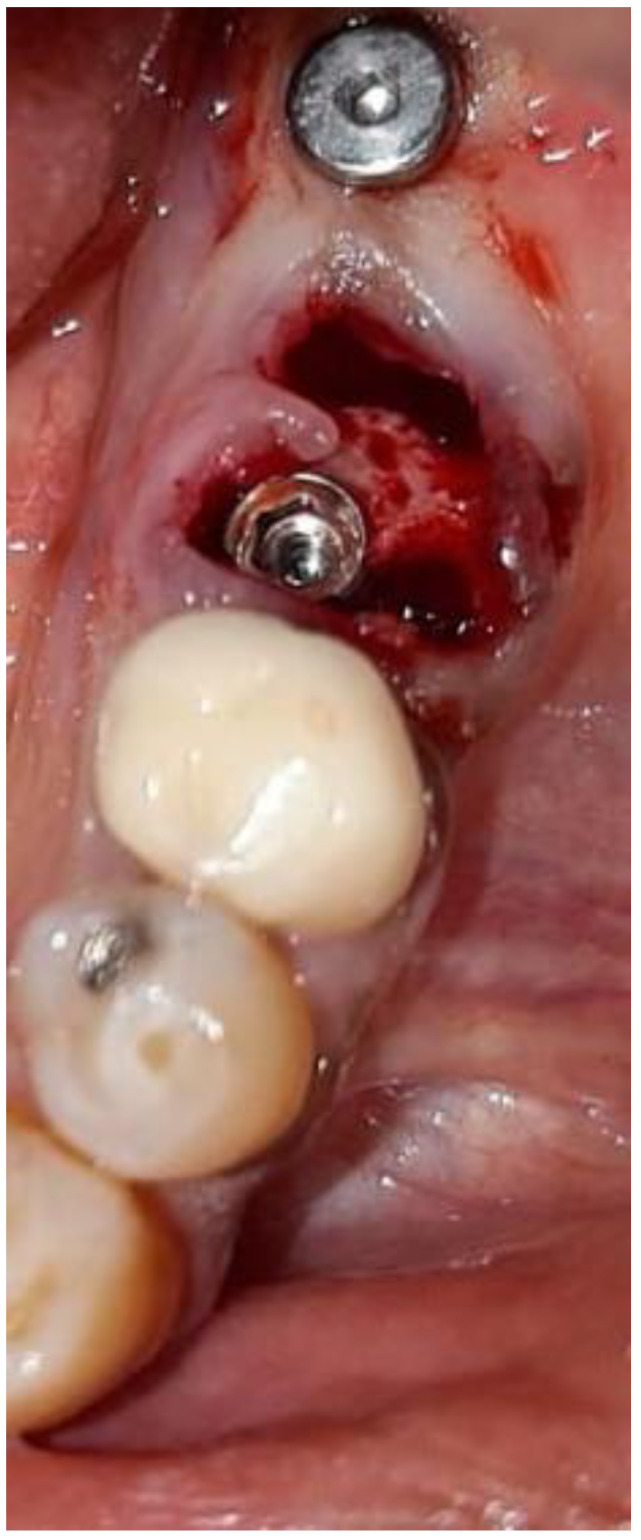
Immediate implant placement; positioning of 4.2 mm wide and 10 mm long dental implant at left mesial root of the first mandibular molar.

**Figure 7:**
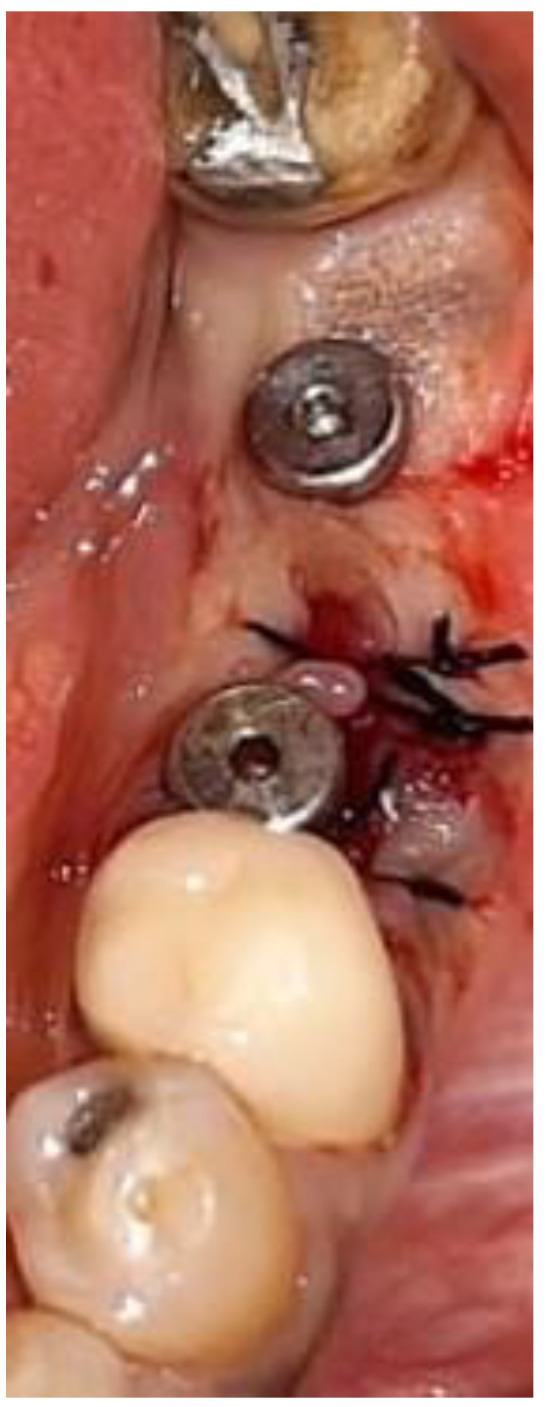
Placement of a 3 mm high healing abutment above a dental implant 4.2 mm wide and 10 mm long positioned at the mesial root of left mandibular first molar. Soft tissue suturing with silk 3–0.

The mean potential RCE was 96% in the second premolar root position using an implant 5mm in diameter; 94% in the first molar root position using an implant 4–4.2 mm in diameter; and 99% in the second molar root position using an implant 4.5–4.8 mm in diameter. With an implant of 5mm diameter, the dental surgeon could predicatively achieve RCE rates of 96%, 99%, and 100% in the second premolar, first molar, and second molar root positions, respectively (Table 1). In Figure 6 and Figure 7, IIP of a 4.2 mm wide and 10 mm long dental implant at the left mesial root of the first mandibular molar is shown. Following implant placement, a 3 mm high healing abutment was positioned and soft tissue sutured with silk 3-0. 

#### 3.2.2. Vertical Aspect

The mean vertical distances for each tooth type ranged from 11.46 mm for the left second molar to 13.99 mm for the left second premolar. The mean (SD) vertical distance values were 13.87 mm (1.87) for second premolars, 12.61 mm (1.86) for first molars, and 11.83 mm (1.73) for second molars (Table 2).

#### 3.2.3. Buccolingual Aspect

The mean buccolingual distance at a point for each root type ranged from 4.3 mm for the left second premolar roots to 6.4 mm for the mesial roots of the right first molar. The mean distance at X point for each root type ranged from 7.0 mm for the left second premolar roots to 9.0 mm for the mesial roots of the left second molar. The mean buccolingual distance values at A and X points for each root type were too wide to support a dental implant of proper diameter.

### 3.3. Modifiers

The potential effect of patient demographic factors on the ability to achieve circumferential bone engagement without passing the apex in IIP was examined.

#### 3.3.1. Sex

The CBCT scans of the 51 female patients included measurements of 402 roots (245 mandibular teeth): 88 (21.9%) second premolar roots, 150 (37.3%) first molar roots, and 164 (40.8%) second molar roots. The distribution of the teeth/roots by patient sex is shown in Table 3. There were no statistically significant differences in mean measurements in the mesiodistal or vertical aspects between the groups.

#### 3.3.2. Age

The study group included 63 patients aged 40 years or less and 37 patients older than 40 years. The CBTC of the younger group included 534 roots of 326 mandibular teeth: 118 (22.1%) second premolar roots, 198 (37.1%) first molar roots, and 218 (40.8%) second molar roots. The distribution of the teeth/roots by patient age variables is shown in Table 3. Patients aged younger than 40 years had a higher mean mesiodistal distance (3 mm vs. 3.4 mm) and, consequently, lower RCE, but the between-group difference was not statistically significant. The younger group had a significantly higher mean vertical measurement (5.7 mm vs. 6.8 mm, *p* < 0.001) and, consequently, a lower AIL (*p* < 0.005, *t*-test and Wilcoxon test). The chance of achieving bone contact without passing the apex was significantly lower in the younger group.

## 4. Discussion

IIP in the posterior mandible, where esthetics is not a major concern, has proven to be a predictable surgical procedure [25], with excellent survival rates of above 94.9% [2,3,4,5,6]. Nevertheless, due to the inferior alveolar canal position and submandibular fossa concavity, bone availability may be limited in the vertical aspect. This can potentially lead to such complications as partial or permanent paresthesia, hematoma, excessive bleeding, and infection [26,27,28,29,30].

Trying to overcome this hindrance, Shah et al. [22] assessed the amount of septal bone available for stabilization of the IIP in posterior mandible. The authors performed 3D alveolar bone assessment of mandibular first molars and found that in 76% of the examined sites, septal bone width was inadequate (mean interradicular bone width, 3.04 mm), compromising the chances for primary stability [22]. Therefore, they proposed that two narrow implants be used to replace one mandibular first molar, assuming that this would avoid an irregularly shaped crown with a cantilevered portion resulting from placing one implant. Thus, for a mesiodistal distance of 12 mm, there would be 1.5 mm wide space between each implant and tooth and a 3 mm wide space between implants, leaving 3.5 mm for each dental implant. These findings suggested that by using two narrow implants, dental practitioners can provide better prosthetic stability and prevent rotational forces on the prosthetic components [22].

However, in the present study, we found that mean RCE values in the root socket were low when a narrow implant (3.3 mm diameter) was placed (59% in the second molar, 70% in the first molar, and 19% in the second premolar) and that increasing the dental implant diameter would make it possible for the dental surgeon to achieve better predictable RCE rates. Corresponding RCE values would be 88%, 94%, and 58% using a 4–4.2 mm implant and 99%, 97%, and 88% using a 4.5–4.8 mm implant.

Regarding the implant length suitable for a single standing implant IIP, 10 mm was found to be the minimum for sufficient bone anchorage that could handle posterior mandible occlusal forces [31]. However, a recent study reported that in 24% of the examined sites, the IAN-to-furcation length was less than 10 mm, warranting vertical bone augmentation or the use of short implants of 8 mm or 6 mm [24]. Short implants should be used adjacent to other implants to allow for splinting and to achieve more durability [23]. We found that the mean AIL for a 10 mm long implant was high and predictable: 99% in the second premolar root position, 90% in the first molar root position, and 86% in second molar root position. For 8 mm and 6 mm implant lengths, the values were even higher for all tooth types (Table 2). Thus, the oral surgeon should opt for a length of 10 mm when placing a single implant and a length of 6 mm or 8 mm when placing multiple adjacent implants, following clinical assessment and CBCT evaluation.

We found a significant difference in mean vertical measurements in all roots between patients aged more or less than 40 years (5.7 mm vs. 6.8 mm, *p* < 0.001). Thus, the chances of achieving bone contact without passing the apex may be lower in younger patients. Given the findings of the present study, we speculate that the vertical distance of the posterior mandible roots decreases with patient age. There may be several reasons for this change, such as periodontal disease leading to alveolar bone loss, occlusal wear, and compensatory eruption over time [32].

The strengths of this study were the virtual placement of dental implants and various detailed measurement points. All measurements were performed twice, and 18% were performed three times, to ensure reliable and repeatable outcomes. The major limitations of the study were our basing the evaluation on pre-extraction CBCTs, without consideration of the trauma sustained by the alveolar bone following extraction. We also did not take into account the specific periodontal status of the patients, the bone quality of the specific assessed site, and other factors affecting osteointegration such as osteoporosis and immunocompromised status. Future studies are needed to explore these factors and their potential impact on achieving a stable IIP. The findings will make it possible to validate the accuracy of the results presented in this study and to evaluate their clinical contribution.

## 5. Conclusions

In the majority of cases, circumferential engagement in the posterior mandible can be achieved during IIP without further apical drilling by using the proper dental implant diameter. The ability to predict primary implant stability can help dental surgeons avoid potential complications associated with apical drilling.

## Figures and Tables

**Table 1:** Mesiodistal distance values and predicted prevalence of radiological circumferential bone engagement by implant diameter.

Tooth Type/Root	Mesiodistal Distance (mm), Mean (SD)				Predicted Prevalence of Radiological Circumferential Engagement by Implant Diameter					
	No. Teeth (Roots)	A Point	F Point	X Point (for Tooth)	Implant Diameter					
					3.3 mm	3.754.1 mm	4–4.2 mm	4.5–4.8 mm	5 mm	5.5 mm
Second molar	156(312)	3.11(0.5)	4.32(0.6)	9.19(0.58)	59%	77%	88%	99%	100%	100%
37D	79	3.3(0.49)	4.67(0.48)	9.25(0.57)	42%	64%	82%	96%	99%	99%
37M	79	2.91(0.44)	4.1(0.47)	9.25(0.57)	82%	90%	93%	99%	100%	100%
47D	77	3.34(0.45)	4.66(0.5)	9.12(0.6)	37%	64%	78%	100%	100%	100%
47M	77	2.87(0.41)	3.86(0.49)	9.12(0.6)	77%	91%	99%	100%	100%	100%
First molar	147(294)	2.98(0.5)	4.41(0.56)	9.14(0.71)	70%	87%	94%	97%	99%	100%
36D	74	3.03(0.41)	4.59(0.46)	9.14(0.78)	67%	88%	97%	99%	100%	100%
36M	74	2.74(0.41)	4.25(0.46)	9.14(0.78)	88%	95%	99%	99%	100%	100%
46D	73	3.23(0.53)	4.76(0.6)	9.14(0.63)	46%	78%	88%	94%	97%	100%
46M	73	2.9(0.51)	4.06(0.43)	9.14(0.63)	78%	88%	94%	97%	99%	100%
Second premolar	175(175)	3.64(0.51)		5.41(0.68)	19%	39%	58%	88%	96%	99%
35	83	3.61(0.55)		5.46(0.77)	21%	43%	64%	88%	95%	99%
45	92	3.66(0.48)		5.37(0.59)	18%	35%	54%	88%	97%	100%

**Table 2:** Vertical distance values by tooth type and prevalence of. predicted implant length available without passing the apical bone.

Tooth Type/Root	Vertical Distance (mm), Mean (SD)		Prevalence of Predicted Available Implant Length		
	No. Teeth (Roots)	X Point (for Tooth)	Implant Length		
			10 mm	8 mm	6 mm
Second molar	156 (312)	11.83 (1.73)	86%	97%	100%
37D	79	11.46 (1.74)	84%	95%	99%
37M	79	12.29 (1.72)	91%	97%	100%
47D	77	11.5 4 (1.72)	82%	97%	100%
47M	77	12.03 (1.78)	87%	100%	100%
First molar	147(294)	12.61 (1.86)	90%	98%	100%
36D	74	12.1 (1.96)	86%	96%	100%
36M	74	13.07 (1.83)	95%	97%	100%
46D	73	12.21 (1.86)	88%	99%	100%
46M	73	13.07 (1.8)	92%	100%	100%
Second premolar	175 (175)	13.8 7(1.87)	99%	100%	100%
35	83	13.99 (1.78)	99%	100%	100%
45	92	13.76 (1.94)	98%	99%	100%

**Table 3:** Distribution of teeth/roots by side, patient age, and patient sex.

Tooth Type/Root	All	Side		Sex		Age (year)	
		Right	Left	Female	Male	≤40 y	40+ y
Total teeth	478	242	236	245	233	326	152
Total roots	781	394	387	402	379	534	247
Number of roots by tooth type							
2nd premolars	175	92	83	88	87	118	57
1st molars	294	148	146	150	144	198	96
2nd molars	312	154	158	164	148	218	94

## Abbreviations

AIL = available implant length, BLT = bone level tapered, CBCT = cone beam computed tomography, IAN = inferior alveolar nerve, IIP = immediate implant placement, RCE = radiological circumferential engagement, SD = standard deviation.

## References

[1] Buser D., Chappuis V., Belser U.C., Chen S. (2017). Implant placement post extraction in esthetic single tooth sites: When immediate, when early, when late?. Periodontology.

[2] Cosyn J., De Lat L., Seyssens L., Doornewaard R., Deschepper E., Vervaeke S. (2019). The effectiveness of immediate implant placement for single tooth replacement compared to delayed implant placement: A systematic review and meta-analysis. J. Clin. Periodontol..

[3] Ketabi M., Deporter D., Atenafu E.G. (2016). A systematic review of outcomes following immediate molar implant placement based on recently published studies. Clin. Implant Dent. Relat. Res..

[4] Lang N.P., Lui P., Lau K.Y., Li K.Y., Wong M.C. (2012). A systematic review on survival and success rates of implants placed immediately into fresh extraction sockets after at least 1 year. Clin. Oral Implants Res..

[5] Mello C.C., Lemos C.A.A., Verri F.R., Dos Santos D.M., Goiato M.C., Pellizzer E.P. (2017). Immediate implant placement into fresh extraction sockets versus delayed implants into healed sockets: A systematic review and meta-analysis. Int. J. Oral Maxillofac. Surg..

[6] Moy P.K., Nishimura G.H., Pozzi A., Danda A.K. (2016). Single implants in dorsal areas—A systematic review. Eur. J. Oral Implantol..

[7] Altintas N.Y., Taskesen F., Bagis B., Baltacioglu E., Cezairli B., Senel F.C. (2016). IIP in fresh sockets versus implant placement in healed bone for full-arch fixed prostheses with conventional loading. Int. J. Oral Maxillofac. Surg..

[8] Werbitt M.J., Goldberg P.V. (1992). The immediate implant: Bone preservation and bone regeneration. Int. J. Periodontics Restor. Dent..

[9] Chan H.L., Benavides E., Yeh C.Y., Fu J.H., Rudek I.E., Wang H.L. (2011). Risk assessment of lingual plate perforation in posterior mandibular region: A virtual implant placement study using cone-beam computed tomography. J. Periodontol..

[10] Chrcanovic B.R., de Carvalho Machado V., Gjelvold B. (2016). Immediate implant placement in the posterior mandible: A cone beam computed tomography study. Quintessence Int..

[11] Leong D.J., Chan H.L., Yeh C.Y., Takarakis N., Fu J.H., Wang H.L. (2011). Risk of lingual plate perforation during implant placement in the posterior mandible: A human cadaver study. Implant Dent..

[12] Smith R.B., Tarnow D.P. (2013). Classification of molar extraction sites for immediate dental implant placement: Technical note. Int. J. Oral Maxillofac. Implants.

[13] Haj Yahya B., Chaushu G., Hamzani Y. (2021). Computed tomography for the assessment of the potential risk following implant placement in fresh extraction sites in the posterior mandible. J. Oral Implantol..

[14] Froum S., Casanova L., Byrne S., Cho S.C. (2011). Risk assessment before extraction for immediate implant placement in the posterior mandible: A computerized tomographic scan study. J. Periodontol..

[15] Lin M.H., Mau L.P., Cochran D.L., Shieh Y.S., Huang P.H., Huang R.Y. (2014). Risk assessment of inferior alveolar nerve injury for immediate implant placement in the posterior mandible: A virtual implant placement study. J. Dent..

[16] Lazzara R.J. (1989). Immediate implant placement into extraction sites: Surgical and restorative advantages. Int. J. Periodontics Restor. Dent..

[17] Lioubavina-Hack N., Lang N.P., Karring T. (2006). Significance of primary stability for osseointegration of dental implants. Clin. Oral Implants Res..

[18] Zitzmann N.U., Naef R., Schärer P. (1997). Resorbable versus nonresorbable membranes in combination with Bio-Oss for guided bone regeneration. Int. J. Oral Maxillofac. Implants.

[19] Zitzmann N., Naef R., Schärer P. (1996). Gesteuerte Knochenregeneration und Augmentation in der Implantatchirurgie mit Bio-Oss und Membrantechniken. Dtsch Zahnärztl Zeitschr..

[20] Misch C.E., Misch C.E. (1999). Root form surgery in the edentulous mandible: Stage I implant insertion. Implant Dentistry-2.

[21] Law C., Alam P., Borumandi F. (2017). Floor-of-mouth hematoma following dental implant placement: Literature review and case presentation. J. Oral Maxillofac. Surg..

[22] Shah Z., Shah A., Raiyani P. (2016). Two narrow implants replacing a mandibular right first molar: A case study. J. Dent. Implants.

[23] Monje A., Fu J.H., Chan H.L., Suarez F., Galindo-Moreno P., Catena A., Wang H.L. (2013). Do implant length and width matter for short dental implants (<10 mm)? A meta-analysis of prospective studies. J. Periodontol..

[24] Padhye N.M., Shirsekar V.U., Bhatavadekar N.B. (2020). Three-dimensional alveolar bone assessment of mandibular first molars with implications for immediate implant placement. Int. J. Periodontics Restor. Dent..

[25] Chen S.T., Wilson T.G., Hämmerle C.H. (2004). Immediate or early placement of implants following tooth extraction: Review of biologic basis, clinical procedures, and outcomes. Int. J. Oral Maxillofac. Implants.

[26] Bartling R., Freeman K., Kraut R.A. (1999). The incidence of altered sensation of the mental nerve after mandibular implant placement. J. Oral Maxillofac. Surg..

[27] Goodacre C.J., Kan J.Y., Rungcharassaeng K. (1999). Clinical complications of osseointegrated implants. J. Prosthet. Dent..

[28] Greenstein G., Tarnow D. (2006). The mental foramen and nerve: Clinical and anatomical factors related to dental implant placement: A literature review. J. Periodontol..

[29] Greenstein G., Cavallaro J., Romanos G., Tarnow D. (2008). Clinical recommendations for avoiding and managing surgical complications associated with implant dentistry: A review. J. Periodontol..

[30] Lamas Pelayo J., Peñarrocha Diago M., Martí Bowen E., Peñarrocha Diago M. (2008). Intraoperative complications during oral implantology. Med. Oral Patol. Oral Cir. Bucal..

[31] Nunes L.S., Bornstein M.M., Sendi P., Buser D. (2013). Anatomical characteristics and dimensions of edentulous sites in the posterior maxillae of patients referred for implant therapy. Int. J. Periodontics Restor. Dent..

[32] Kerr N.W., Ringrose T.J. (1998). Factors affecting the lifespan of the human dentition in Britain prior to the seventeenth century. Br. Dent. J..

